# Transcriptional Response of Polycomb Group Genes to Status Epilepticus in Mice is Modified by Prior Exposure to Epileptic Preconditioning

**DOI:** 10.3389/fneur.2015.00046

**Published:** 2015-03-10

**Authors:** James P. Reynolds, Suzanne F. C. Miller-Delaney, Eva M. Jimenez-Mateos, Takanori Sano, Ross C. McKiernan, Roger P. Simon, David C. Henshall

**Affiliations:** ^1^Department of Physiology and Medical Physics, Royal College of Surgeons in Ireland, Dublin, Ireland; ^2^Department of Neurosurgery, Mie University School of Medicine, Tsu, Mie, Japan; ^3^Morehouse School of Medicine, Atlanta, GA, USA

**Keywords:** epileptic tolerance, hippocampal sclerosis, neuroprotection, temporal lobe epilepsy, polycomb

## Abstract

Exposure of the brain to brief, non-harmful seizures can activate protective mechanisms that temporarily generate a damage-refractory state. This process, termed epileptic tolerance, is associated with large-scale down-regulation of gene expression. Polycomb group (PcG) proteins are master controllers of gene silencing during development that are re-activated by injury to the brain. Here, we explored the transcriptional response of genes associated with polycomb repressive complex (PRC) 1 (*Ring1A*, *Ring1B*, and *Bmi1*) and PRC2 (*Ezh1*, *Ezh2*, and *Suz12*), as well as additional transcriptional regulators *Sirt1*, *Yy1*, and *Yy2*, in a mouse model of status epilepticus (SE). Findings were contrasted to changes after SE in mice previously given brief seizures to evoke tolerance. Real-time quantitative PCR showed SE prompted an early (1 h) increase in expression of several genes in PRC1 and PRC2 in the hippocampus, followed by down-regulation of many of the same genes at later times points (4, 8, and 24 h). Spatio-temporal differences were found among PRC2 genes in epileptic tolerance, including increased expression of *Ezh2*, *Suz12*, and *Yy2* relative to the normal injury response to SE. In contrast, PRC1 complex genes including *Ring 1B* and *Bmi1* displayed differential down-regulation in epileptic tolerance. The present study characterizes PcG gene expression following SE and shows prior seizure exposure produces select changes to PRC1 and PRC2 composition that may influence differential gene expression in epileptic tolerance.

## Introduction

Epigenetic processes are structural modifications to chromatin which are indicative of and contribute to the transcriptional state (1). These processes include DNA methylation and histone modification and they play an important role in gene expression control. Such modifications are dynamic in the adult brain and may act as important transcriptional determinants in plasticity and memory (2, 3). Aberrant DNA methylation has been implicated in certain neurological disorders (4, 5), and other studies have emphasized the role of epigenetic mechanisms in seizures and epilepsy (6). For instance, increased levels of enzymes regulating DNA methylation has been reported in human temporal lobe epilepsy (TLE) (7), which may have important effects on gene expression (8). Further, changes in histone acetylation (at promoter elements of *Gria2*, *Bdnf*, *c-fos*, and *Creb*) and altered histone deacetylase (HDAC) activity have been noted after experimental status epilepticus (SE) (9–11) and possibly human TLE (12). Changes in the activity of transcriptional repressor RE1-silencing transcription factor (REST) also play a role in events associated with seizures (13), likely through altered histone modification (14).

Polycomb group (PcG) proteins are a large conserved family of transcriptional repressors (15). Originally described in *Drosophila melanogaster* as key silencers of Hox genes, they assemble as polycomb repressive complexes (PRCs) at the chromatin, regulating its structure and altering transcriptional activity through histone modification and effector recruitment (16–18). Deregulation of developmentally silenced genes through alteration of PcG signaling has been observed in various malignancies (19), while their contribution to lineage specification during neurogenesis is well established (20–22). Despite observations of dynamic PcG activity in postmitotic neurons (23, 24), few studies have addressed the role of PcG-mediated repression in neurological disease, and none in epilepsy. Derepression of PcG targets may be involved in l-DOPA-induced dyskinesia (25) and ischemic excitotoxicity (26). PcG-mediated repression has also been implicated in ischemic tolerance (27). This phenomenon shares many characteristics with epileptic tolerance (28, 29), where brief seizures activate a coordinated response of gene expression changes that render brain tissue refractory to subsequent insults that would otherwise be damaging (28, 30). Protection can be independent of changes to seizure severity during SE (29), likely represents the recruitment of active neuroprotective mechanisms and long-lasting changes in gene expression (28, 30) and is accompanied by a reduction in the number of spontaneous seizures evolving after SE (29).

The molecular mechanisms regulating altered gene expression in epileptic tolerance are not fully understood. Previous work suggested transcription factors such as NFκB (31) and AP1 (32) may drive gene synthesis-dependent tolerance, consistent with observations of wide-ranging divergences in gene transcription (29) and gene methylation (33) between epileptic injury (non-preconditioned animals) and epileptic tolerance (preconditioned animals). Given that epileptic tolerance is associated with a coordinated suppression of excitability- and excitotoxicity-related genes (29) and CpG island, hypermethylation is more common in tolerance that in injury (33), it seems likely that transcriptional repression is also a key modality of epileptic tolerance. Here, we performed an extensive spatio-temporal characterization of PcG transcript expression following SE, comparing responses between non-preconditioned and preconditioned mice.

## Materials and Methods

### Animal procedures

All animal experiments were carried out in accordance with guidelines outlined in the European Communities Council Directive (86/609/EEC) and the European Union Directive (2010/63/EU). All experimentation was approved by the Research Ethics Committee of the Royal College of Surgeons in Ireland (REC #205) and performed under license from the relevant authority [Department of Health, Dublin, Ireland (license number B100/4423)]. Adult male C57BL/6 mice, aged 6–10 weeks (20–30 g), were obtained from Harlan (UK) and housed in a climate-controlled biomedical facility on a 12 h light/dark cycle with food and water provided *ad libitum*.

Focal-onset SE was induced by intra-amygdala (i.a.) stereotaxic microinjection of kainic acid (KA) as described previously (34). Briefly, mice were anesthetized using isoflurane (5% induction, 1.5–2% maintenance) under normothermic conditions and placed in a stereotaxic frame (Stoelting Co.). A midline scalp incision was made, Bregma located and a craniectomy performed (stereotaxic coordinates: AP = −0.95 mm; L = −2.85 mm). Next, a guide cannula was placed over the dura and the assembly fixed by dental cement. Animals were then removed from the frame and placed in an open-top container that allowed free movement for recordings. Microinjection of KA [3.75 mm subdural depth; 1 μg in 0.2 μL phosphate buffered saline (PBS)] (Sigma-Aldrich, Dublin, Ireland) was carried out in awake mice. Non-seizure control mice received injection of vehicle alone. Mice received lorazepam (6 mg/kg, intraperitoneal, i.p.) 40 min following i.a. injections to curtail seizures, reduce mortality, and restrict cerebral damage. Seizure preconditioning was accomplished by i.p. injection of KA (15 mg/kg) 24 h prior to SE induced by i.a. KA (29). Control and injury (i.e., non-preconditioned) mice were sham-preconditioned with i.p. saline.

Mice were euthanized between 1 and 24 h following i.a. injections. Saline-perfused whole brains were fresh-frozen in 2-methylbutane at −30°C and sectioned on a cryostat (12 μm) prior to immunostaining. Hippocampal subfield microdissection (of CA3-, CA1-, and DG-enriched portions) was carried out as previously described (35). In brief, following bisection of isolated whole brain and removal of the cerebellum and midbrain, the hippocampal structures were separated and removed after placing a curved forceps between the hippocampal fimbria and the lateral ventricle. The isolated tissue was rolled out over the cortex and extraneous tissue was removed, yielding intact whole hippocampus. The hippocampus was microsurgically partitioned along a longitudinal axis using fine curved forceps, with the CA1 located in the superior/posterior partition. Microdissections were immediately stored at −80°C.

### Gene expression analyses

All tissue was homogenized in Trizol (Qiagen, West Sussex, UK) and RNA was isolated using a standard extraction method. Briefly, following homogenization, chloroform-mediated phase separation and isopropanol-mediated precipitation were carried out. RNA was washed and reconstituted in RNase-free H_2_O. All nucleic acid extract concentrations were determined using a NanoDrop 2000 (Thermo Scientific, Reading, UK) prior to reverse transcription (RT). RNA extracts were treated with DNAse1 (Invitrogen, Dublin, Ireland) to eliminate contaminating genomic DNA and normalized to between 500 ng and 1 μg prior to RT. RT was performed using random hexamer primers (Fermentas, York, UK) and Superscript II reverse transcriptase (Invitrogen). Following RT, qPCR was carried out on a Lightcycler 2.0 (Roche, Sussex, UK) using Quantitect SYBR Green PCR kits (Qiagen) and custom designed primers (Primer 3.0, Sigma-Aldrich) for target genes. The following primer sequences were used: *Bmi1*, forward TGTCCAGGTTCACAAAACCA and reverse TGCAACTTCTCCTCGGTCTT; *Ring1a*, forward CCTGGACATGCTGAAGAACA and reverse TCCCGGCTAGGGTAGATTTT; *Ring1b*, forward ACGGACCAAAACCTCTGATG and reverse AGTGGCATTGCCTGAAGTCT; *Ezh2*, forward GGCTAATTGGGACCAAAACA and reverse GAGCCGTCCTTTTTCAGTTG; *Ezh1*, forward CTCAGTGGCAACATGCCTAA and reverse CCCACAAACACAACCAACAG; *Suz12*, forward AGAAAACGAAATCGCGAAGA and reverse CGTTGGTTTCTCCTGTCCAT; *Yy1*, forward TGAGAAAGCATCTGCACACC and reverse CGCAAATTGAAGTCCAGTGA; *Yy2*, forward GCCTCTTTTACGGGCTTTCT and reverse ACCATCGATCTGCTTCTGCT; *Sirt1*, forward GCCTGTTGAGGATTTGGTGT and reverse TAAATTTGGGGGCAATGTTC; β-actin, forward GGGTGTGATGGTGGGAATGG and reverse GGTTGGCCTTAGGGTTCAGG. β-actin was used for the normalization of mRNA expression levels. Of those PcG genes for which transcript variants exist (*Ezh2*, *Suz12*, and *Sirt1* code for two isoforms each), primers targeted regions common to both isoforms. PCR products for *Bmi1*, *Ring1a*, *Ring1b*, *Ezh2*, *Suz12*, and *Yy1* spanned exon–exon junctions, while those for *Ezh1* and *Sirt1* targeted the 3′UTR. The PCR product for *Yy2* (having just one exon) was derived from within the coding sequence. Non-reverse transcribed extracts and non-template reactions were used as negative controls. For assessment of basal transcription levels of PcG subunits, both the ΔCT value (versus the CT value of β-actin) and the 2^−ΔΔCT^ (or RQ) value (versus a reference ΔCT value derived from the mean ΔCT value for all PcG transcripts) were plotted. Significant differences between subfields for each PcG subunit were computed using the comparative cycle threshold method (2^−ΔΔCT^, normalized against the average ΔCT value of the CA3). For investigation of SE-induced changes in PcG transcription, the comparative cycle threshold method was again employed to assess the relative fold change in target transcript levels for each PcG subunit (versus the average ΔCT value of time-matched control samples). In parallel, primer specificity was investigated using Taq Polymerase PCR in a Veriti Thermocycler (Applied BioSystems, Warrington, UK). Amplification products were run in a 2% agarose gel (100 V, 15 min, with 1:10000 ethidium bromide) and imaged in a FujiFilm LAS-3000 (Fuji, Sheffield, UK) under UV (312 nm, 0.125 s exposure). For RT-qPCR analyses comparing control, injury and tolerance, three groups of mice (control, injury, and tolerance, *n* = 4) were used. For those analyses looking at differences in basal PcG expression between transcripts, control mice from across all four sampled timepoints were grouped (*n* = 16), as sham surgery did not alter basal PcG transcription in the hippocampus (Figure S1 in Supplementary Material). These samples (ipsilateral CA3, CA1, and DG) were independent of those used in immunohistochemistry and western blotting analyses.

### Subcellular fractionation

Subcellular fractionation was undertaken to examine protein localization in nuclear and cytosolic compartments. Ipsilateral hippocampal tissue was pooled (two hippocampal dissections per lysate) and homogenized in M-SHE buffer [210 mM mannitol, 70 mM sucrose, 10 mM HEPES-KOH pH 7.4, 1 mM EDTA, 1 mM EGTA, and a protease inhibitor cocktail (P8340, Sigma-Aldrich)]. A nuclear-enriched pellet was isolated by repeated centrifugation and trituration (two spin cycles at 1200 × *g*, 10 min at 4°C); the supernatant, containing a crude mix of mitochondrial, microsomal, and cytoplasmic constituents, was processed as detailed below. Following further trituration and centrifugation (1000 × *g*, 10 min at 4°C), the nuclear fraction was resuspended in TSE buffer (10 mM Tris pH 7.5, 300 mM sucrose, and 1 mM EDTA) with 0.1% NP-40. A pellet bilayer emerged upon further centrifugation (8600 × *g*, 10 min at 4°C), whereby the upper opaque layer was retained and purified through repeated centrifugation. The resulting pellet, representing a nuclear-enriched fraction, was resuspended in lysis buffer and analyzed through SDS-PAGE. A crude cytoplasmic fraction was isolated and purified using repeated high-speed centrifugation steps (two spins at 1200 × *g*, 10 min; two spins 10,000 × *g*, 15 min; one spin 16,000 × *g*, 5 min; at each stage the pellet was discarded and the upper 4/5 of supernatant retained). Fraction quality was determined using immunoblotting against subcellular fraction-specific markers (Lamin A/C, nucleus; GAPDH, cytoplasm).

### Western blotting

Western blotting was performed on hippocampal subfield microdissections, whole hippocampus, cortex, and cerebellum. Tissue was homogenized in SDS-lysis buffer (150 mM NaCl, 50 mM Tris-HCl, 1 mM EDTA, 1% NP-40, pH 8.0) or M-SHE lysis buffer. All lysis buffers included protease and phosphatase inhibitor cocktail (1:100, Cat P8340, Sigma-Aldrich) or individually added volumes of PMSF (1:500), aprotinin (1:1000), leupeptin (1:1000), and vanadate (1:1000). Homogenates were spun (14,000 × *g*, 10 min at 4°C) and the supernatant was retained. Nucleus-containing pellet was also retained and later resuspended in SDS-lysis buffer for certain applications. Protein concentration was determined using the micro BCA protein assay (Pierce, Rockford, IL, USA). Lysates were boiled at 95°C in gel-loading buffer and separated by SDS-PAGE (4/6–15%), before being electroporated onto nitrocellulose or PVDF membranes. Membranes were incubated overnight at 4°C with primary antibodies against: trimethyl-H3K27 (1:500), EZH2 (1:500), RING1A (1:1000), RING1B (1:1000), BMI1 (1:400), SUZ12 (1:1000), and Lamin A/C (1:1000), all rabbit polyclonal (Cell Signaling Technology, Danvers, MA, USA). Band visualization was obtained through incubation with secondary horseradish peroxidase-conjugated antibodies (20°C, Jackson Immuno-Research, Suffolk, UK), followed by Super Signal West Pico chemiluminescent substrate (Pierce). Images were captured using a FujiFilm LAS-4000 (Fuji). Densitometry was performed using ImageJ. Briefly, chemiluminescent density plots for each sample were generated and the area of the region of interest (corresponding to the expected molecular weight) was normalized to the matching loading control (Lamin A/C). For western blotting analyses, ipsilateral hippocampus from three groups of mice (control, injury, and tolerance) were used. For whole cell lysates, *n* = 5 mice per group were assessed. For analysis of nuclear-enriched fractions, *n* = 3 samples were used following pooling of two hippocampal isolates per sample. Animals were from an independent cohort to those used in RT-qPCR and immunohistochemistry analyses.

### Immunohistochemistry

Immunostaining was performed on fresh-frozen coronal sections (12 μm) prepared at the level of medial (1.9–2.1 mm posterior of Bregma) hippocampus. Sections were air-dried, fixed in 4% PFA solution, and processed for immunostaining using antibodies [anti-EZH2, anti-Trimethyl-H3K27, anti-NeuN (mouse monoclonal, Millipore, Cork, Ireland)], in 5% goat serum/0.1% Triton-X in PBS. Immunoreactivity was visualized using Alexa Fluor 488 and 568 secondary antibodies (Molecular Probes, OR, USA). Sections were labeled with Hoechst nuclear stain to visualize nuclei. Tissue was mounted in FluorSave (Sigma-Aldrich). All images were captured using a Nikon 2000s epifluorescence microscope. Control mice only (*n* = 3) were used for these analyses. Animals were from an independent cohort to those used in RT-qPCR and western blotting analyses.

### Statistics

All data and statistical analyses were carried out using Microsoft Excel, Graphpad Prism, and Stata. Comparisons were made using one-way analysis of variance, followed by Tukey *post hoc* testing. Significance was accepted at *P* < 0.05. All data are presented as mean ± SEM.

## Results

### Polycomb repressive complex component expression in the adult mouse hippocampus

**Figure 1:**
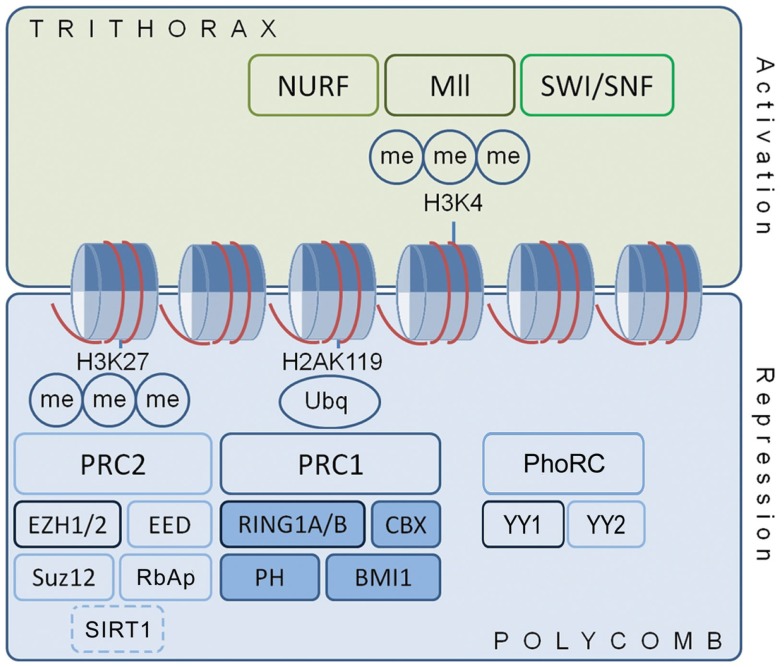
**Polycomb and trithorax proteins regulate chromatin structure and gene expression**. Cartoon shows polycomb protein complex organization in relation to transcription. PcG proteins regulate chromatin structure through histone tail methylation and other post-translational modifications. PcG proteins often localize about discrete genetic elements with the functionally antagonist trithorax proteins, a diverse group of transcriptional activators, to yield a bivalent, “primed” state that is amenable to rapid alteration in response to signaling events. Ancillary DNA-binding proteins, such as YY1 of the Pho Repressive Complex, aid recruitment of PRC1 and PRC2 to target genes. They also interact with other DNA and chromatin modifiers, including DNMTs, histone deacetylases, histone acetyltransferases, and the Jarid pathway proteins. H2A and H3, histone 2A and histone 3; Me, methyl group; PRC, polycomb repressive complex; Ubq, ubiquitin.

Previous studies have reported expression of particular PcG members within the adult brain (21, 36, 37), but there has not been a comprehensive characterization of the expression of PcG transcripts and proteins in the hippocampus of the adult C57BL/6 mouse. Figure 1 shows the key genes forming the PRCs. The two major polycomb complexes described, defined by the major catalytic constituent, are PRC1 (containing RING1A and 1B) and PRC2 (containing EZH2). A third complex, known as the PhoRC (containing Yin Yang 1, YY1), may also be involved (15, 19). Transcriptional repression depends on the coordinated action of these complexes, which may display significant redundancies (38).

**Figure 2:**
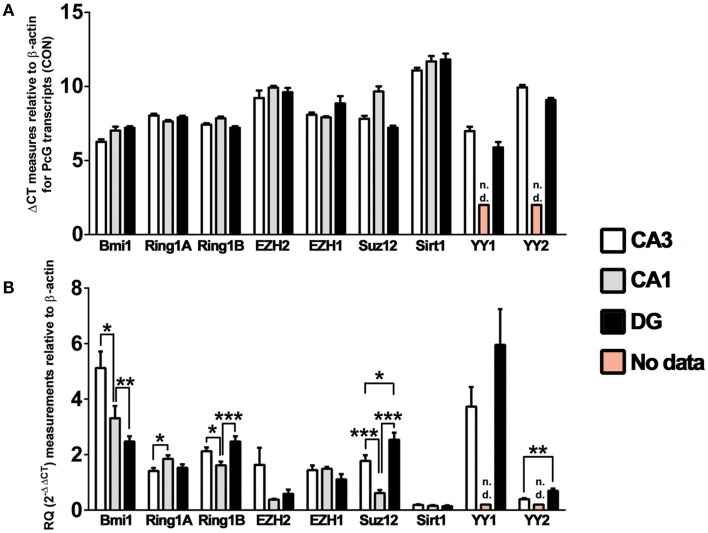
**Polycomb group transcripts expression in the mouse hippocampus**. Graphs show qRT-PCR data and both the ΔCT and RQ for various core PcG transcripts, relative to β-actin. Measurements of basal PcG transcription, expressed as **(A)** the ΔCT value and **(B)** a derived RQ value (2^−ΔΔCT^ versus the mean ΔCT value for all transcripts and subfields) were made using hippocampus from mice that received intra-amygdala PBS injection (*n* = 16), with analysis performed on microdissected hippocampal fractions enriched for CA3, CA1, and DG. Derived RQ values in **(B)** were statistically appraised by one-way analysis of variance to determine differences between subfields in basal transcription of each PcG subunit. Data expressed as mean ± SEM. *P < 0.05, **P < 0.01, ***P < 0.001 for *n* = 16. CA, *cornu ammonis*; CON, control; DG, dentate gyrus; n.d., no data.

In order to generate a comparative profile of basal PcG expression in the hippocampus, we performed RT-qPCR on microdissected hippocampal subfields (CA3-, CA1-, and DG-enriched fractions) and calculated the difference in cycle threshold (ΔCT) value against β-actin in vehicle-injected control mice (used for subsequent investigations of polycomb regulation following SE). The transcripts analyzed were; *Bmi1*, *Ring1a*, and *Ring1b* of PRC1; *Ezh2*, *Ezh1*, *Suz12*, and *Sirtuin 1* (*Sirt1*) of PRC2; and *Yy1* and *Yy2* of the PhoRC; chosen on the basis of their roles in polycomb function, previous implications in neuronal function taken from the literature, and the availability of other resources for further study, including antibodies. It was found that all nine PcG transcripts analyzed were expressed and detectable in the hippocampus. ΔCT values for the expression of these transcripts in control mice were plotted for CA3, CA1, and DG (*n* = 16, Figure 2A). Qualitative assessment suggested that *Bmi1* is the highest expressed PcG transcript across all subfields, while *Sirt1* is the lowest. *Yy1* expression was higher than *Yy2* expression in both the CA3 and the DG, and in CA3 and CA1, *Ezh1* expression exceeded *Ezh2* expression. *Ring1a* and *Ring1b* were expressed at similar levels.

We then quantitatively assessed differences in expression of PcG transcripts between subfields. Again, using pooled data across all timepoints in control mice (*n* = 16), we calculated the RQ for ΔΔCT values normalized to the mean ΔCT value for all transcripts and subfields (Figure 2B). Normalizing to single ΔCT value ensured that all summary data in this figure were relatable. These analyses suggest that *Bmi1* is expressed significantly higher in the CA3 than in the CA1, which in turn was higher than the DG. Of the other constituents of PRC1, *Ring1a* appears to be higher in CA1 than CA3, while *Ring1b* is lowest in CA1, suggesting subfield-specific regulation of PcG components. Concerning PRC2, only *Suz12* appears differentially expressed between subfields, with significantly lower expression in CA1 when compared to CA3 and DG. Finally, *Yy2* of the PhoRC is higher in DG than in CA3. In order to account for potential effects arising from surgery- and cannula-associated manipulation prior to PBS injection, we plotted ΔCT values for each timepoint (*n* = 4) and confirmed that time elapsed after PBS injection was not likely to contribute to the observed ΔCT values (Figure S1 in Supplementary Material).

**Figure 3:**
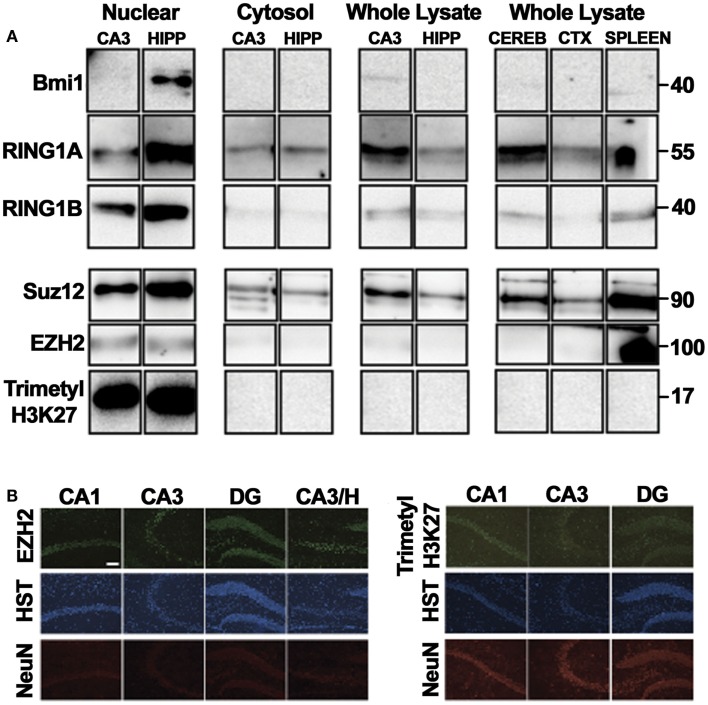
**Principal polycomb group proteins in the mouse hippocampus**. Western blot depictions here were obtained following multiple exposures. For nuclear and cytosolic fractionation, either three CA3 tissue samples or two hippocampal tissue samples were pooled and run numerous times for each immunoblot. For analysis of whole lysates, lysates were from a single animal. Each column represents one lysate, with each row representing an independent immunoblot and exposure. **(A)** Western blotting was performed on whole cell lysates of the CA3, whole hippocampus, cerebral cortex, cerebellum, and spleen as well as cytosolic and nuclear fractions of the CA3 and the whole hippocampus to confirm the expression of PcG proteins in the adult brain. Observed molecular weights based on the migration of a protein ladder are given (right). **(B)** The neuronal expression of the polycomb repressive complex 2 (PRC2) catalytic subunit EZH2 was validated by immunofluorescence, with a similar pattern of expression observed for the PRC2-associated epigenetic histone modification, trimethylation of lysine27 in histone 3. CA, *cornu ammonis*; CEREB, cerebellum; CTX, cortex; DG, dentate gyrus; H, hilus; trimethyl-H3K27, trimethylated lysine 27 of histone 3; HIPP, hippocampus; HST, Hoechst. Scale, 50 μm.

Protein levels of six PcGs were investigated by western blotting using subcellular fractions (Figure 3A). For three of the six, protein was only observed in the nucleus. Cytosolic expression was apparent for the remaining three proteins, though it appeared significantly lower in comparison to nuclear levels (Figure 3A), in keeping with functional roles in regulating transcription. We suspect that the reason some proteins are not detected in whole lysate preparations is that recovery of the nuclear fraction is poor using a general homogenization procedure.

Immunofluorescence microscopy was also performed to assess the spatial expression pattern of Ezh2 and trimethyl-histone 3 lysine 27 (trimethyl-H3K27), the characteristic histone modification catalyzed by PcG proteins. In each case, nuclear-specific immunoreactivity in neuronal populations was observed throughout the hippocampal subregions (CA3, CA1, DG, and hilus), as well as in certain non-specified glial cells (Figure 3B), as supported by Hoechst stain and NeuN immunoreactivity.

### Status epilepticus induces rapid, distinct changes in polycomb transcript levels

Next, a spatio-temporal expression profile of several major PcG transcripts within the hippocampus was established for mice given either i.a. vehicle (control), i.a. KA with sham-preconditioning (injury) or i.a. KA with preconditioning (tolerance). The main site of injury in this model is the CA3 subfield, with minor cell death observed in the CA1 and DG regions. As before (29), animals preconditioned by low-grade seizure activity 24 h earlier displayed approximately 50% less hippocampal damage compared to injury mice after an equally severe episode of SE (data not shown).

**Figure 4:**
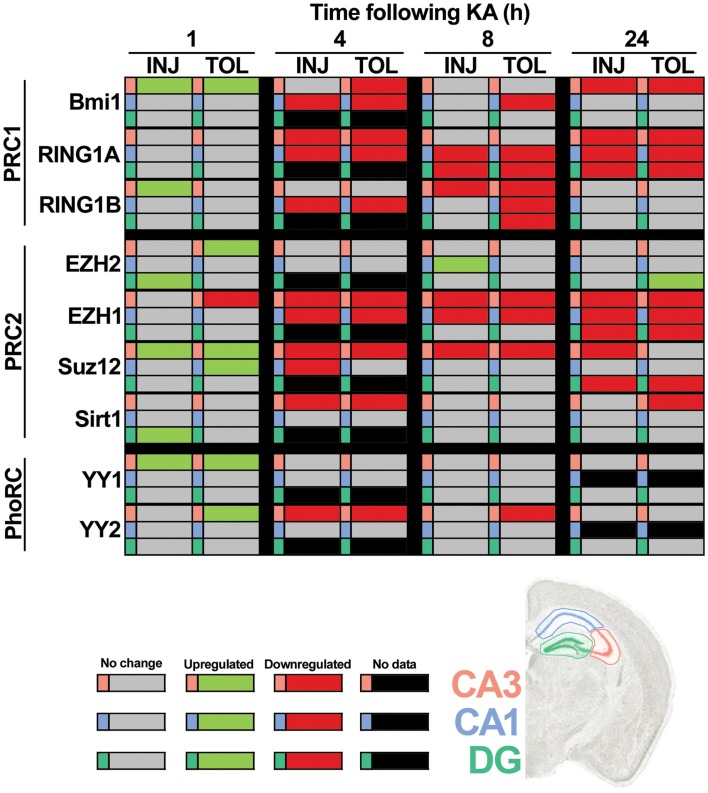
**Focal-onset SE causes rapid changes in polycomb transcript levels in the hippocampus of sham-preconditioned and seizure-tolerant mice**. Relative polycomb transcript expression, as compared to control and corrected to β-actin, was determined using qRT-PCR. All significant differences are indicated in green (up-regulation) or red (down-regulation), as determined versus control following one-way analysis of variance with Tukey *post hoc* test. Gray cells indicate no significant difference versus control samples and black cells indicate that no data are available for that particular cell. Polycomb members/complexes are indicated and tabulated by timepoint (1, 4, 8, and 24 h), experimental group (epileptic injury and tolerance), and hippocampal subfield (CA3, CA1, and DG). CA, *cornu ammonis*; DG, dentate gyrus; INJ, injury; KA, kainic acid; PRC, polycomb repressive complex; TOL, tolerance.

**Figure 5:**
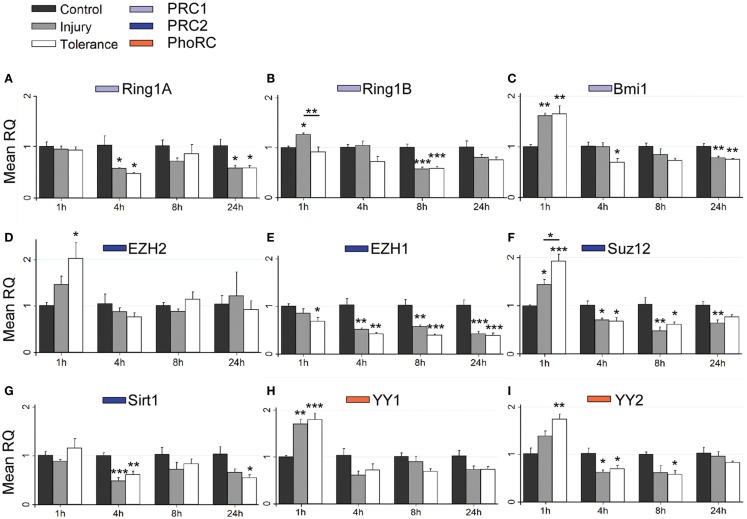
**Focal-onset SE causes rapid changes in polycomb transcript levels in the CA3 of sham-preconditioned and seizure-tolerant mice**. **(A–I)** Relative fold expression of various PcG mRNA transcripts was measured in the CA3 using qRT-PCR in vehicle-injected (control), sham-preconditioned (injury), and seizure-tolerant (tolerance) mice at 1, 4, 8, and 24 h following SE. Expression was corrected to β-actin and normalized to control (*n* = 4). *P < 0.05, **P < 0.01, ***P < 0.001 (versus control unless indicated), one-way analysis of variance with Tukey *post hoc* test. PRC, polycomb repressive complex.

**Figure 6:**
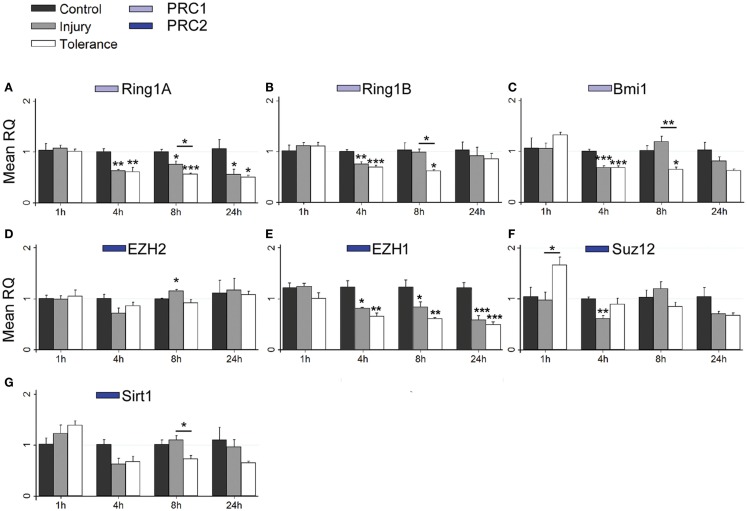
**Focal-onset SE causes rapid changes in polycomb transcript levels in the CA1 of sham-preconditioned and seizure-tolerant mice**. **(A–G)** Relative fold expression of various PcG mRNA transcripts was measured in the CA1 using qRT-PCR in vehicle-injected (control), sham-preconditioned (injury), and seizure-tolerant (tolerance) mice at 1, 4, 8, and 24 h following SE. Expression was corrected to β-actin and normalized to control (*n* = 4). *P < 0.05, **P < 0.01, ***P < 0.001 (versus control unless indicated), one-way analysis of variance with Tukey *post hoc* test. PRC, polycomb repressive complex.

**Figure 7:**
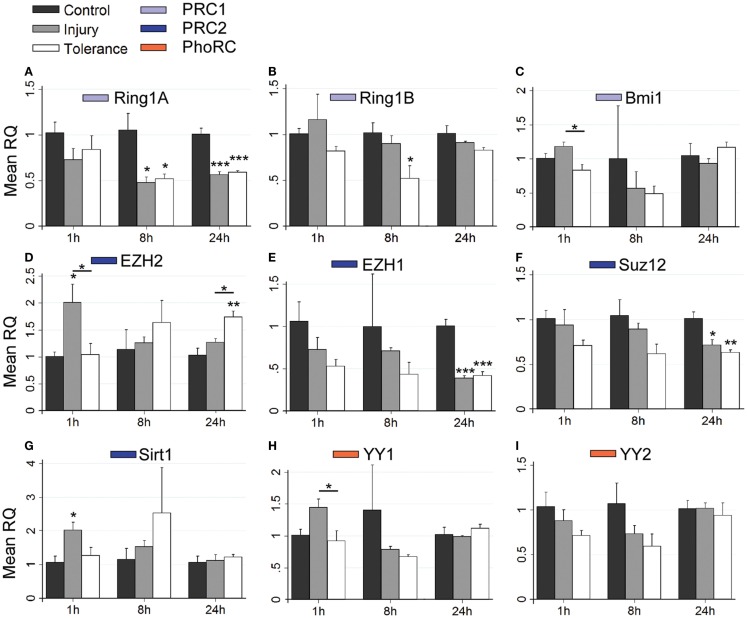
**Focal-onset SE causes rapid changes in polycomb transcript levels in the DG of sham-preconditioned and seizure-tolerant mice**. **(A–I)** Relative fold expression of various PcG mRNA transcripts was measured in the DG using qRT-PCR in vehicle-injected (control), sham-preconditioned (injury), and seizure-tolerant (tolerance) mice at 1, 8, and 24 h following SE. Expression was corrected to β-actin and normalized to control (*n* = 4). *P < 0.05, **P < 0.01, ***P < 0.001 (versus control unless indicated), one-way analysis of variance with Tukey *post hoc* test. PRC, polycomb repressive complex.

Using RT-qPCR, we quantified levels of nine principal PcG members within hippocampal subfields at time points up to 24 h. This included transcripts associated with PRC1 (*Ring1a*, *Ring1b*, and *Bmi1*), PRC2 (*Ezh2*, *Ezh1*, *Suz12*, and *Sirt1*) and the PhoRC (*Yy1* and *Yy2*). RT-qPCR data are summarized in Figure 4, with significant up-regulation (green) or down-regulation (red) indicated, as compared to control. Individual subfield data are presented for CA3 (Figures 5A–I), CA1 (Figures 6A–G), and DG (Figures 7A–I). Analyses show that SE induced rapid bidirectional changes in mRNA levels of various PcG transcripts in CA3, CA1, and DG, with down-regulation the broad response for both injury and tolerance.

Considering changes after SE, there was pronounced down-regulation of *Ring1a* in all subfields, between 4 and 24 h (Figures 5A, 6A, and 7A). *Ring1b* levels were decreased at 4 h in CA1 and 8 h in all subfields before recovering to baseline at 24 h (Figures 5B, 6B, and 7B). *Bmi1* was down-regulated in CA3 and CA1 between 4 and 24 h (Figures 5C and 6C).

For PRC2 complex genes, we observed significant down-regulation of *Ezh1* at 4, 8, and 24 h across all subfields (Figures 5E, 6E, and 7E). In contrast, no instances of *Ezh2* down-regulation were observed (Figures 5D, 6D, and 7D). Similarly to *Ezh1*, *Suz12* was down-regulated in CA3 at 4, 8, and 24 h, as well as at 4 h in CA1 and 24 h in DG (Figures 5F, 6F, and 7F). *Sirt1* down-regulation was confined to CA3 at 4 h (Figure 5G). Finally, *Yy2* of the PhoRC was decreased at 4 and 8 h post-SE in the CA3 only (Figure 5I). In general, down-regulation seemed more prevalent in the CA3, but was seen in DG and CA1 at later timepoints between 4 and 24 h (Figure 4). However, down-regulation of *Sirt1* and *Yy2* was specific to the CA3 at all timepoints (Figure 4). The number of down-regulation events in the CA3/CA1/DG was 1/0/0 (1 h), 11/9/0 (4 h), 7/6/3 (8 h), and 8/4/6 (24 h), respectively, suggesting that the extent of down-regulation peaked at 4 h in the CA3 and CA1, but was greatest at 24 h in the DG. In contrast, up-regulation was seen to occur dominantly at 1 h (12 of 14 analyzed), and was generally restricted to the CA3 (9 of 12 such events). Levels of *Ring1b* (Figure 5A) and *Bmi1* (Figure 5C) of PRC1 were increased at 1 h, while up-regulation of *Ezh2* (Figures 5D, 6D, and 7D), *Suz12* (Figures 5F and 6F), and *Sirt1* (Figure 7G) of PRC2 was observed. *Yy1* and *Yy2*, of the PhoRC, were also seen to be significantly higher at 1 h (Figures 5H,I). With the exception of *Ezh2*, no PcG transcript was increased at 4 h or later following SE.

### Differential expression of PcG genes in epileptic tolerance

**Figure 8:**
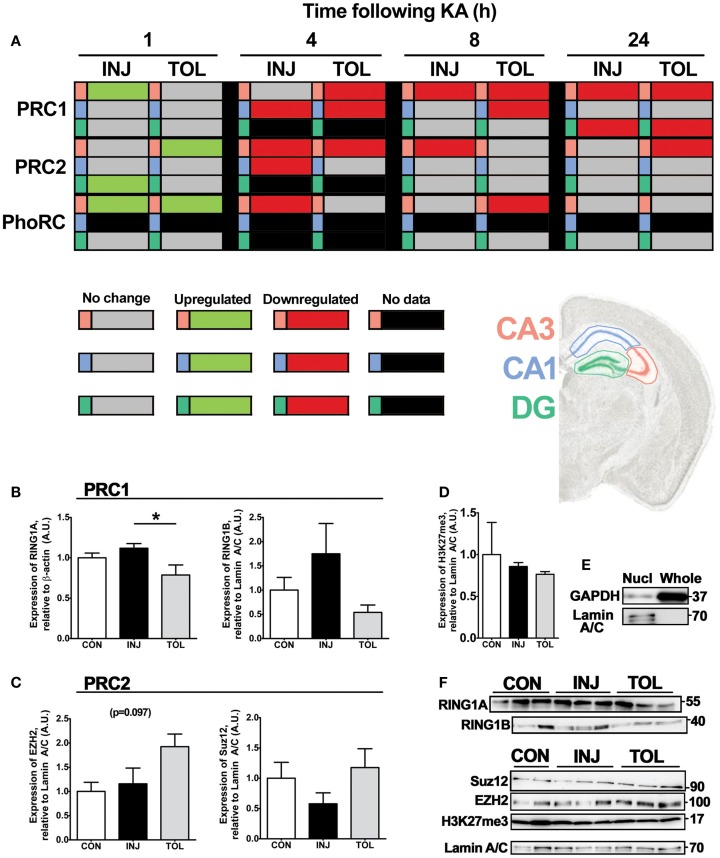
**Expression of individual PRC constituents following SE predicts complex-wide effects in the hippocampus**. **(A)** Relative polycomb repressive complex score was calculated using the mean RQ value for member constituents determined using qRT-PCR, as compared to control and corrected to β-actin. All significant differences are indicated in green (up-regulation) or red (down-regulation) as determined versus control following one-way analysis of variance with Tukey *post hoc* test. Gray cells indicate no significant difference versus control samples and black cells indicate that no data are available for that particular cell. Polycomb complexes are indicated and tabulated by timepoint (1, 4, 8, and 24 h), experimental group (epileptic injury and tolerance), and hippocampal subfield (CA3, CA1, and DG). **(B–F)** Semi-quantification of **(B)** RING1A and RING1B, **(C)** EZH2 and SUZ12, and **(D)** trimethyl-H3K27 was performed using western blotting, relative to the endogenous controls β-actin (for RING1A) or Lamin A/C (all others), in whole or nuclear-fractionated lysates of the hippocampus (*n* = 3–5). **(E)** Markers of cellular compartments Lamin A/C and GAPDH were eluted for nuclear-enriched and whole cell lysates. Representative blots used in semi-quantification analysis are depicted in **(F)**, *n* = 1 per lane. Observed molecular weights based on the migration of a protein ladder are shown (right). Data expressed as mean ± SEM in **(B–D)**. *P < 0.05, one-way analysis of variance. CA, *cornu ammonis*; CON, control; DG, dentate gyrus; H3K27me3, trimethylated lysine 27 of histone 3; INJ, injury; KA, kainic acid; Nucl, nuclear-enriched lysate; PRC, polycomb repressive complex; TOL, tolerance.

Seizure preconditioning had relatively modest effects on SE-induced changes in PcG transcript expression with notable exceptions. For transcripts of PRC1 components, decreases were more associated with tolerance than injury. *Ring1b* down-regulation at 8 h was hippocampus-wide in tolerance but restricted to the CA3 in injury (Figures 5B, 6B, and 7B). *Ring1b* up-regulation at 1 h, meanwhile, was injury-specific (Figure 5B), and further, there was tolerance-specific down-regulation of *Bmi1* at 4 h (CA3, Figure 5C) and 8 h (CA1, Figure 6C). Notably, there was no instance of injury-specific down-regulation or tolerance-specific up-regulation for transcripts of the PRC1. Conversely, down-regulation of *Suz12* (of PRC2) was more associated with injury.

*Suz12* up-regulation was seen in the CA3 and CA1 of tolerance mice at 1 h but was restricted to CA3 in injury and at lower levels than seen in tolerance (Figures 5F and 6F). Down-regulation of *Suz12* at 4 h (CA1, Figure 6F) and 24 h (CA3, Figure 5F) was injury-specific. There were also minor differences seen with *Sirt1* and *Yy2* (see Figure 4). Finally, *Ezh2* was seen to increase in both seizure groups in a time- and subfield-specific manner (Figures 5D, 6D, and 7D); at 1 h, up-regulation was specific to the DG in injury, but was restricted to the CA3 in tolerance. *Ezh2* was also increased at 8 and 24 h, in the CA1 of injury mice (8 h only, Figure 6D) and in the DG of seizure-tolerant mice (24 h only, Figure 7D). Interestingly, *Ezh2* up-regulation in the CA3 at 1 h in tolerance was coupled to a tolerance-specific down-regulation of *Ezh1* in CA3 at 1 h (Figure 5E).

### Expression of individual PRC components after status epilepticus reflects complex-wide effects in the hippocampus

To determine if down-regulation of PcG transcripts aligned with those associated with the same PRC, the mean RQ per complex was calculated using RQ scores for each of the PRC constituents analyzed, across the CA3, CA1, and DG hippocampal subfields. These scores revealed that expression of individual PRC constituents predicts complex-wide effects in the hippocampus. Following transient up-regulation at 1 h following SE, scores for PRC1, PRC2, and PhoRC demonstrate coordinated down-regulation of polycomb transcription from 4 h onward, with some divergence between injury and tolerance (summarized in Figure 8A; see also Figure S2 in Supplementary Material). As with individual components, down-regulation of PRC1 was more extensive in tolerance than with injury. For instance, in the CA3, there was a significant increase in PRC1 scores in injury at 1 h (*P* < 0.01, compared to control, Figure 8A; also Figures S2A,B in Supplementary Material) and a tolerance-specific decrease in PRC1 score at 4 h in tolerance (*P* < 0.05, compared to control, injury). PRC1 down-regulation also appeared to be more widespread throughout the hippocampus when considering tolerance, with numerous decreases at 4 and 8 h following SE (Figure 8A). Conversely, PRC2 down-regulation was more prevalent at 4 and 8 h after SE in injury groups, with injury-specific decreases in PRC2 score in CA1 (4 h, *P* < 0.05, compared to control, Figure 8A; also Figures S2C,D in Supplementary Material) and CA3 (8 h, *P* < 0.05, compared to control, Figure 8A; also Figures S2A,B in Supplementary Material). As with individual constituents, changes in PcG transcription in the DG were minimal before 24 h (Figure 8A; also Figures S2E,F in Supplementary Material). In sum, it was apparent that divergences between injury and tolerance concerned either up-regulation (at 1 h) or diminished down-regulation (between 4 and 24 h) of specific PRCs, where increased PRC1 score was associated with injury and increased PRC2 score was associated with tolerance. There were no observed divergences between injury and tolerance in the DG at 8 or 24 h (Figure 8A; also Figures S2E,F in Supplementary Material). A full breakdown of significance is shown in Figures S2B,D,F in Supplementary Material.

Given this association of changes in PRC component transcription with either injury or tolerance, we analyzed protein levels at 24 h following SE, when preceding changes in mRNA expression might be expected to manifest. Through western blotting and subcellular fractionation, SE-mediated effects on PcG protein expression were assessed in injury and tolerance mice. Assessment included the use of whole cell protein lysates as well as nuclear fractions, isolated from whole hippocampus. Elution of nuclear-specific Lamin A/C is shown (Figure 8E). For nuclear fractionation, hippocampal samples were pooled (two hippocampal samples per lysate). We assessed protein levels of RING1A and RING1B of PRC1, EZH2, and SUZ12 of PRC2 and trimethyl (Lys27)-Histone 3, the polycomb-associated chromatin mark (Figures 8B–F). In whole cell lysates, RING1A (PRC1) levels were higher in injury than tolerance at 24 h in the hippocampus (*P* < 0.05, Figures 8B,F). In nuclear-enriched fractions, there were no significant differences in RING1B (PRC1), EZH2 (PRC2), or SUZ12 (PRC2) protein levels (Figures 8B,C,F). There was an apparent threefold increase in RING1B levels in injury compared to tolerance, as well as a twofold increase in both EZH2 and SUZ12 levels in tolerance compared in injury, a PRC-specific alignment that was also seen in transcript analysis. Notably, we did not observe an associated loss of trimethylation of lysine 27 in histone 3 in nuclear-enriched hippocampal lysates at 24 h after SE, suggesting that acute global loss of this modification is not a feature of SE (Figures 8D,F).

## Discussion

Polycomb group proteins are a conserved family of transcriptional silencers which were recently linked to the neuroprotection observed in ischemic tolerance (27). Here, we report the effect of prolonged seizures on PcG expression in the adult mouse hippocampus and how this is altered in the setting of epileptic tolerance. The present study also comprises the first detailed comparison of the relative expression of the various transcripts of the PRCs between hippocampal subfields. We found that in injury-group mice, SE induced rapid, bidirectional changes in levels of PcG family gene expression. We found an almost uniform pattern of rapid up-regulation (1 h) followed by later down-regulation (4 h and thereafter) of various transcripts. Broad down-regulation was seen to continue to 24 h post-SE which may be relevant to gene expression patterns beyond the initial period of cell death in the model (34). The amount of time elapsed after SE is the major determinant of the changes, though there are region-specific changes evident. Alterations in PcG transcript levels were more extensive and more rapid in the CA3 and CA1 than the DG, suggesting that SE-elicited gene expression responses are more pronounced in those populations most vulnerable to damage in this model (29). This is predictable, since transcription would be expected to be reduced in damaged cells. Nevertheless, the finding that all populations displayed reductions in transcription suggests the prolonged seizures, rather than cell death *per se*, is responsible for many changes.

Spatio-temporal-specific changes in gene expression may be a common and important feature of SE. In a comprehensive microarray study conducted across hippocampal subfields and several timepoints, subfield represented a more discriminatory parameter than seizure frequency when comparing changes in gene expression following SE (39). Distinct functional clusters were also associated with each of the sampled timepoints, representing acute, latent, and chronic stages of evoked epilepsy (39). Other microarray studies on individual subfields of the hippocampus including the CA3 (40), the CA1 (41), and the DG (42) recapitulate this general trend, with each study noting clustered expression changes in largely non-overlapping functional groups. In terms of the well-characterized PcG proteins YY1 and SIRT1, such temporal and regional dynamics in expression are also evident. Region-specific expression of YY1 has been reported to underlie differential expression of adenosine A2A receptors in the brain (43). YY1 and its binding partners have also demonstrated stimulus-specific patterns of expression and activity following electroconvulsive shock, KA, and PTZ (44–46). Evoked changes in SIRT1 expression, meanwhile, were noted *in vitro* and *in vivo*, following induced epileptiform activity, SE, and electroconvulsive shock (47–49). Changes to YY1 and SIRT1 were bidirectional and evolved over time, depending on the model and stimulus.

The present study did not explore whether altered PcG expression influences the levels of genes under the control of PRCs. This will require further studies targeting specific PcG genes or entire PRC complexes in the model. We can, however, postulate mechanisms by which altered PcG expression could influence cellular outcomes after SE. In addition to direct control of genes regulating apoptosis, various PcG proteins have been observed to directly bind and modify p53, a protein implicated in seizures and damage after SE. BMI1, for instance, can bind p53 with PRC1 subunits RING1A and RING1B, leading to ubiquitination and degradation of p53 (50). This activity was directly attributed to RING1B, an E3 ubiquitin ligase, where RING1B knockdown sensitized cells to apoptosis (51). BMI1 deficiency is associated with p53 accumulation, Bcl-2 down-regulation, and increased hippocampal apoptosis (52). Sirt1 inhibition of p53 function is well documented (53), while EZH2 has been noted to indirectly modulate p53 signaling and depletion of EZH2 leads to increased apoptosis in response to DNA damage (54). PcG repression has also been linked to REST function. Transcriptional regulation by REST has been implicated in epileptogenesis after SE (13) and REST has been observed to bind and recruit both PRC1 and PRC2 to certain REST target genes (55). Indeed, REST depletion causes loss of trimethyl-H3K27 in murine stem cells, and transgenic insertion of promoter fragments containing REST-binding elements can recruit trimethyl-H3K27 (56).

Changes to PcG expression may influence axonal and dendritic structures, which could also be important after SE and for later development of epilepsy. EZH2 recruitment to the *Bdnf* locus has been implicated in nominal restriction of dendritic arborization and activity-induced BDNF expression was linked to derepression of the *Bdnf* locus following reduced EZH2 binding (24). EZH2 is crucial to the regulation of hippocampal neurogenesis and axonal guidance, with downstream deficits in memory function following conditional knockout (22, 23). Neurogenesis is enhanced following seizures and may contribute to seizure-related pathologies (57) and polycomb as well as trithorax group proteins have been implicated in neurogenic processes (20). Together, the wide-ranging reductions in PcG expression that we observed following SE suggest functional studies could focus on the potential relationship between PcGs and p53 accumulation, alterations in REST, and changes in neuronal morphology or neurogenesis.

A major focus of the present study was the differential expression of PcG family genes in epileptic tolerance. A damage-tolerant state was generated by exposing mice to seizure preconditioning a day before SE. Tolerant mice display only ~50% of the normal damage to the CA3 subfield after SE and previous work has shown the neuroprotection is associated with widespread transcriptional silencing (29). More recently, genome-wide methylation analysis revealed a small number of genes were differentially hypermethylated in tolerance compared to injury (33), suggesting epigenetic processes are important in the protection. PcG proteins, as transcriptional repressors, may contribute to this tolerant phenotype. Indeed, BMI1 and Scmh1 have been implicated as contributing factors in ischemic tolerance through their repressive action on potassium channel proteins (27). Accordingly, a reasonable prediction would have been to see up-regulation of PcG genes in epileptic tolerance. In fact, we generally found down-regulation of PcG transcripts in both injury and tolerance. However, the extent of down-regulation between PRC1 and PRC2 diverged for injury and tolerance. The number of down-regulation events across hippocampal subfields and timepoints was greater for injury than tolerance for PRC2. Changes in protein expression of RING1A and RING1B (of PRC1) and EZH2 and SUZ12 (of PRC2) were concordant with this observation. The possibility of divergences in activity of PRC1 and PRC2 between injury and tolerance is interesting. Although polycomb complexes are generally recruited together in a stepwise fashion, it has been shown that PRC2 can occupy certain genomic sites independently of PRC1 (58), while PRC1 has been shown to be capable of chromatin binding in the absence of PRC2 (59). Given that bivalent domains with recruitment of both complexes may mediate stricter transcriptional silencing (58) and that REST can differentially recruit PRC1 in a context-specific manner (60), SE-induced changes in the balance of PRC1 and PRC2 are likely to have significant effects on transcriptional regulation. This is borne out by observations that differential expression of PRC1 subunit BMI1 and PRC2 subunit EZH2 results in repression of different targets (61).

Given its putative role in the regulation of neuronal morphology and cell death pathways, the apparent increase of EZH2 in tolerance at 24 h is particularly intriguing. We observed that, in control mice, *Ezh1* mRNA expression exceeds that of *Ezh2*, particularly in the CA3 and CA1, in keeping with previous profiles of PcG expression in non-proliferative tissues (62). *Ezh1* expression is profoundly decreased throughout the hippocampus following SE, regardless of preconditioning. Does tolerance-specific up-regulation of *Ezh2* at 24 h following SE offset this *Ezh1* deficiency? EZH1 and EZH2 are homologous members of the PRC2 that may have partially overlapping roles and redundancy. For instance, global K3K27me3 can be preserved by EZH1-containing PRC2 in *EZH2^−/−^* cells, where subsequent depletion of EZH1 leads to translational derepression in these cells (63). Redundancy in EZH1 and EZH2 is supported by other double knockout studies (64), but there is also conflicting data suggesting that EZH2 deficiency is sufficient to destabilize global trimethyl-H3K27 (62). Notably, none of these studies have reported extensive demethylation of H3K27 following EZH1 depletion alone, in keeping with our observations that trimethyl-H3K27 levels are unchanged after SE. However, some sub-functionalization of these proteins is apparent. EZH1 may only target a subset of EZH2 genes and has inferior methyltransferase activity (62). It has been suggested that PRC2-EZH2 is more active in *de novo* H3K27 di- and trimethylation, while PRC2-EZH1 is involved in maintenance of the mark and transcriptional repression (65). Further, EZH1 and EZH2 have different chromatin blinding and compaction properties. Our transcript and protein analysis suggests that tolerance may be associated with an increased retention of EZH2 following widespread loss of *Ezh1* expression. As such, changes to PRC2–EZH2 activity in both injury and tolerance represents an ideal next step in delineating possible transcriptional regulatory changes arising after SE. The lack of destabilization in trimethyl-H3K27 at 24 h after SE suggests that such changes, if any, may be restricted to a small number of sites.

The mechanisms of PcG recruitment to distinct genomic elements are also unclear (15) and it is not yet known whether PRE are widespread in mammalian genomes (15). However, a recent study confirmed that rapid derepression of PcG target genes can occur following a pathological insult in the brain and confirmed several neuronal PcG targets (25). Subsequent investigations involving chromatin immunoprecipitation and proteomic analyses represent a viable means of further delineating the structure, interactions, and targets of PcG complexes in the adult brain.

In summary, we have characterized regional and temporal changes in PcG expression in the adult mouse hippocampus. Our data show that SE produces immediate changes in the transcription of polycomb genes, with divergence noted for preconditioned animals. Functional data are now required to link the observed gene changes for PcG proteins to cell injury and other outcomes after SE in injury and tolerance.

## Conflict of Interest Statement

The authors declare that the research was conducted in the absence of any commercial or financial relationships that could be construed as a potential conflict of interest.

## Supplementary Material

The Supplementary Material for this article can be found online at http://www.frontiersin.org/Journal/10.3389/fneur.2015.00046/abstract
