# Supplementary material for: Transcriptional Response of Polycomb Group Genes to Status Epilepticus in Mice is Modified by Prior Exposure to Epileptic Preconditioning
Source: Front Neurol. 2015 Mar 10;6:46. doi: 10.3389/fneur.2015.00046 (PMC4354380; doi:10.3389/fneur.2015.00046)
Supplement: Supplementary file 1 [file Image_1.PDF]

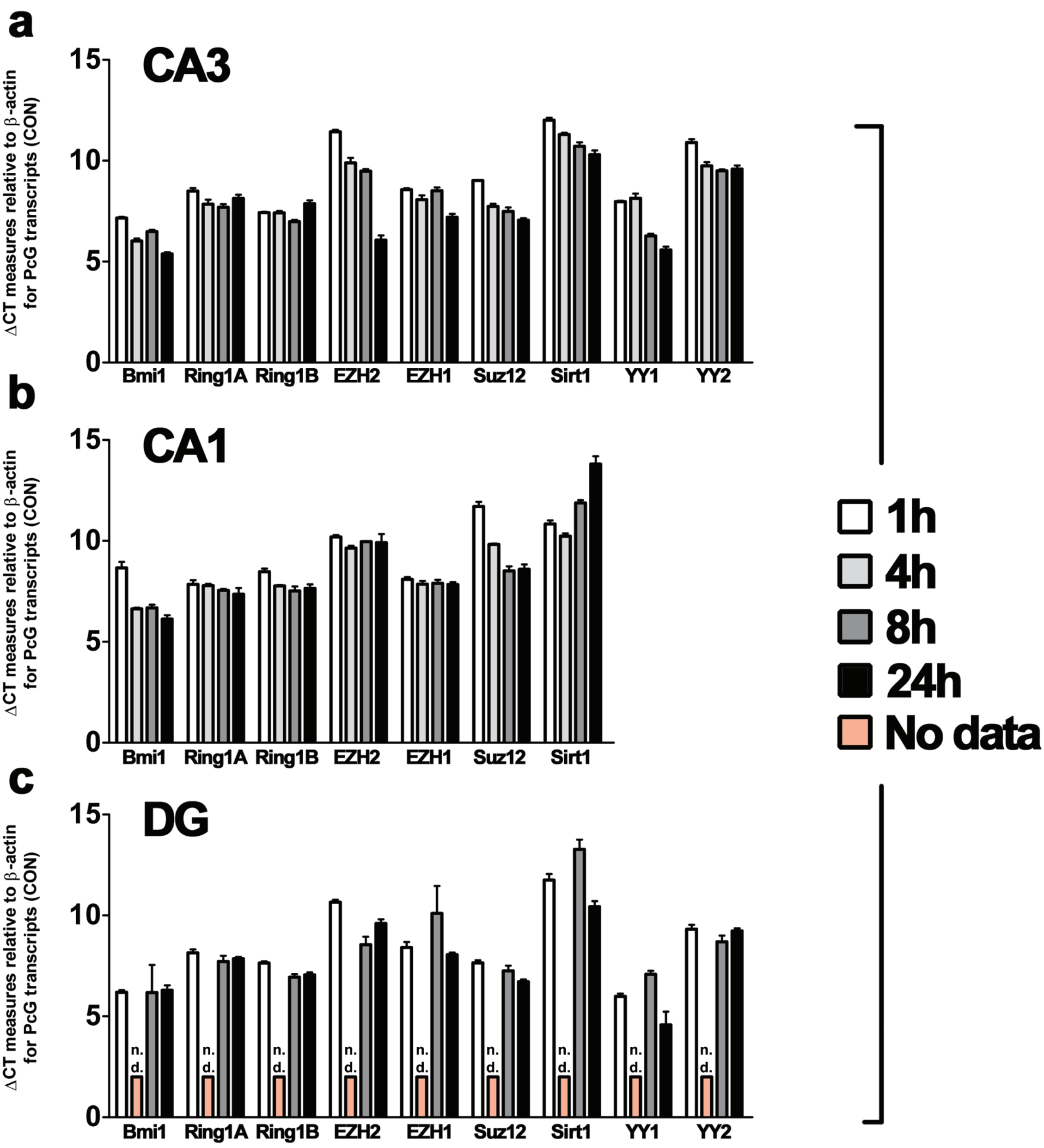

Supplementary Figure 1 | Sham surgery does not alter basal Polycomb Group transcription in the hippocampus. Hippocampal homogenates were analysed by qRT-PCR and the ΔCT for various core PcG transcripts against an endogenous control, β-actin, was plotted. Measurements were from sham-surgery mice receiving intraamygdala injection of PBS (n = 4 for each timepoint) with analysis performed on microdissected hippocampal fractions enriched for **(a)** CA3, **(b)** CA1 and **(c)** DG. CA, cornu ammonis; CON, control; DG, dentate gyrus.; n.d., no data.
