# Supplementary material for: Transcriptional Response of Polycomb Group Genes to Status Epilepticus in Mice is Modified by Prior Exposure to Epileptic Preconditioning
Source: Front Neurol. 2015 Mar 10;6:46. doi: 10.3389/fneur.2015.00046 (PMC4354380; doi:10.3389/fneur.2015.00046)
Supplement: Supplementary file 2 [file Image_2.PDF]

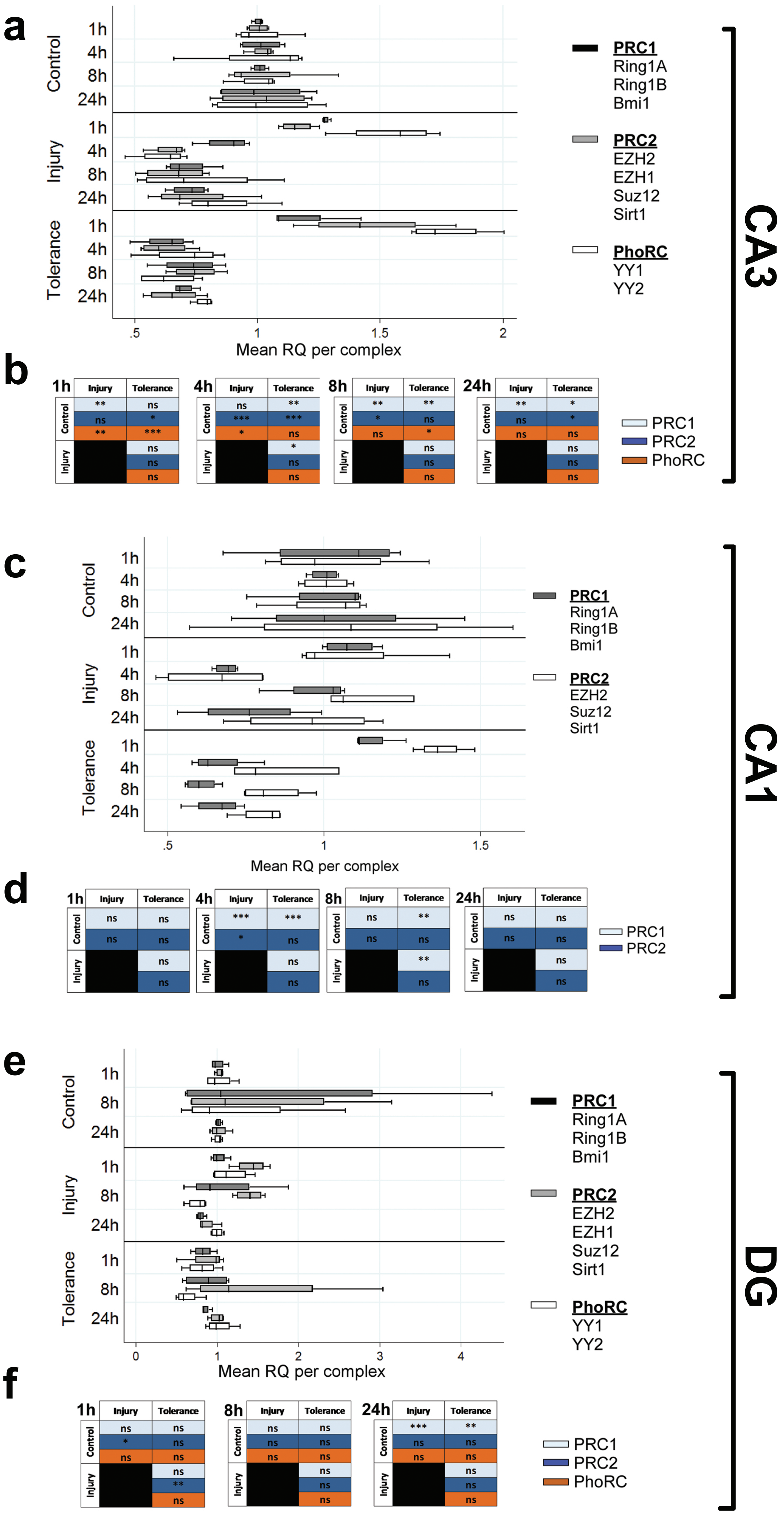

Supplementary Figure 2 | Expression of individual PRC constituents following SE predicts complex-wide effects in the hippocampus. **(a)** Mean RQ scores of CA3 expression for each of the three PcG complex groups were generated using qRT-PCR measurements as in Figures 4-7. Expression was corrected to β-actin (n = 4). **(b)** Statistical outputs demonstrating statistically significant differences in **(a)** were computed using analysis of variance with Tukey post-hoc test. All data expressed as mean ± S.E.M., *P < 0.05, **P < 0.01, ***P < 0.001. Similar analyses are presented for CA1 (**c, d**) and DG **(e,f)**. CA, cornu ammonis; DG, dentate gyrus; PRC, Polycomb Repressive Complex.
